# Supplementary material for: Change in Fatty Acid Composition in High-Temperature-Damaged Rice Grains and Its Effects on the Appearance and Physical Qualities of the Cooked Rice
Source: Foods. 2025 Sep 4;14(17):3097. doi: 10.3390/foods14173097 (PMC12428571; doi:10.3390/foods14173097)
Supplement: Supplementary file 1 [file foods-14-03097-s001.zip › foods-3758148-supplementary.pdf]

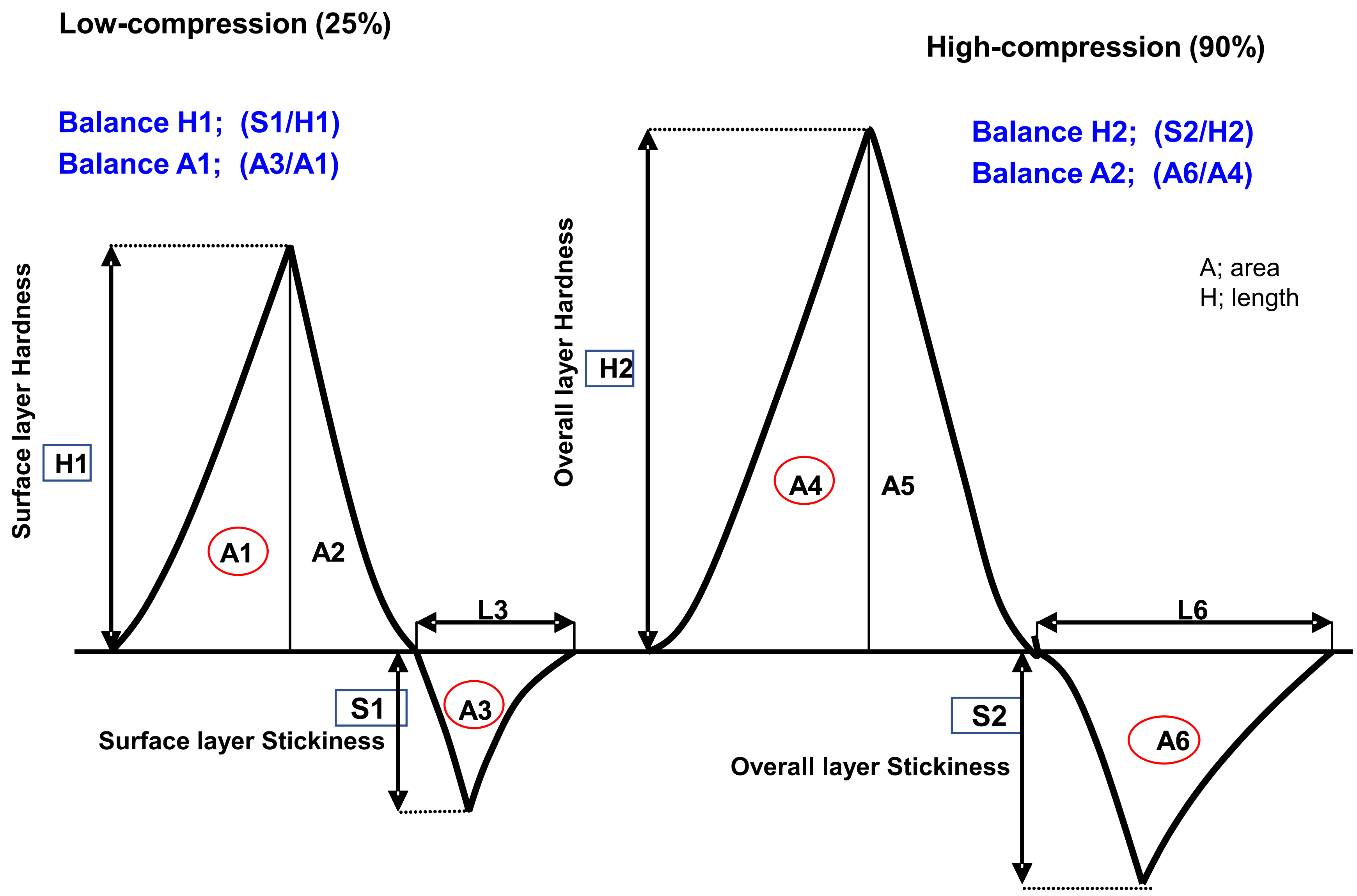

Figure S1: The physical properties of the boiled rice grains by the low-compression (25 %) and high -compression (90 %) methods with the Tensipresser.

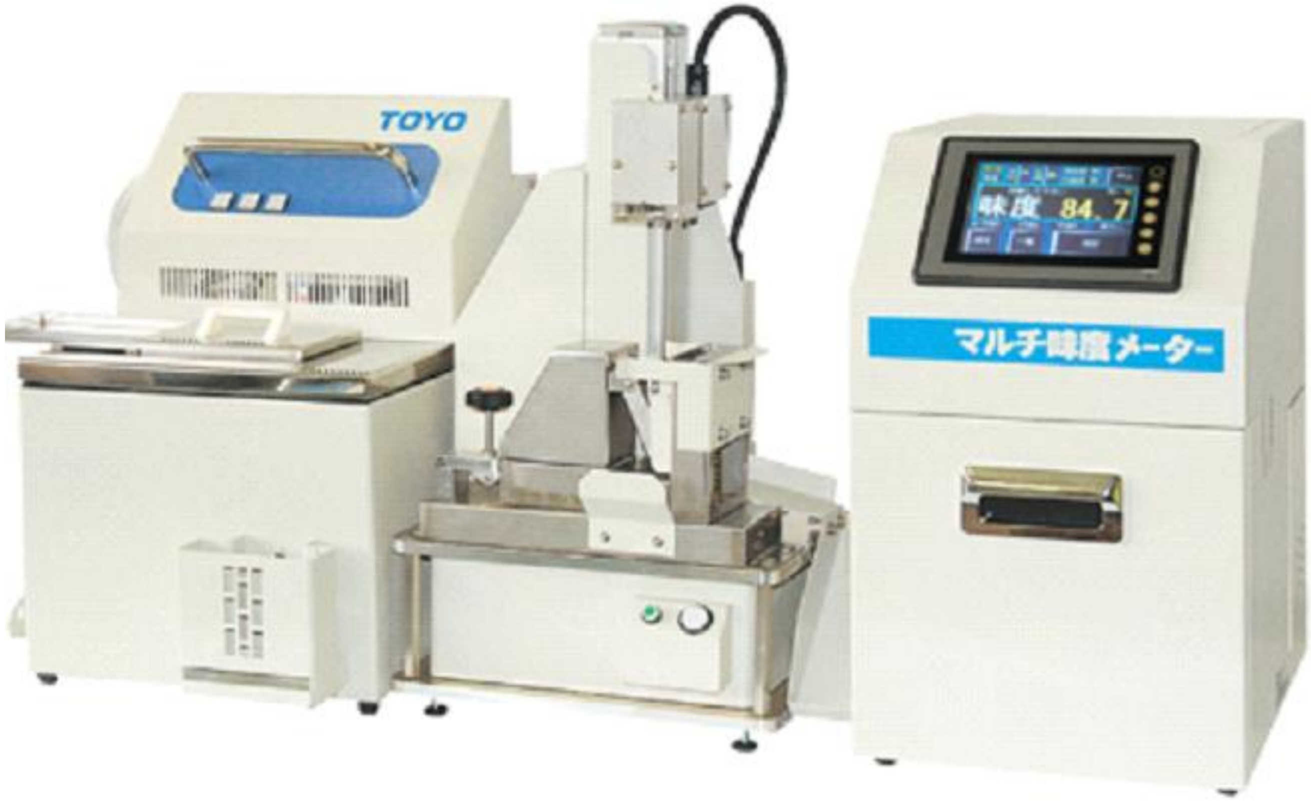

Figure S2, Mido Meter (Toyo Rice Co., Wakayama, Japan)

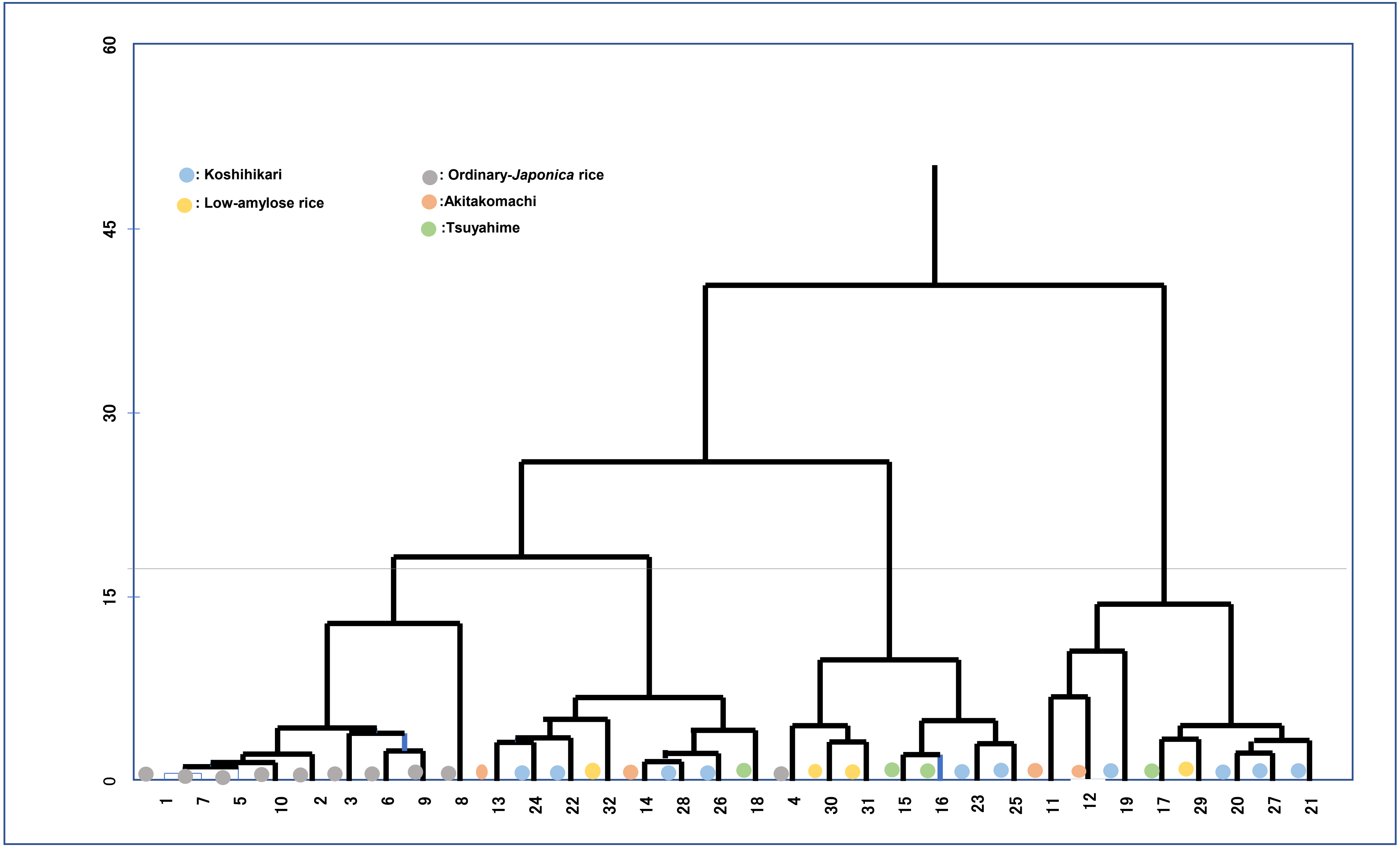

Figure S3, Cluster analysis for 32 samples in 2023 using Pt, taste degree, oleic acid, linoleic acid, palmitic acid and Fb_3(37>DP).

Table S1: α- amylase activities of 32 unpolished rice in 2022.

	α-amylase (CU/g)
Gohyakukawa	0.078 ± 0.011 c
Kazesayaka	0.093 ± 0.001 b
Sasanishiki	0.071 ± 0.008 c
Ginganoshizuku	0.070 ± 0.001 c
Hatsushimo	0.116 ± 0.006 a
Koshiibuki	0.058 ± 0.004 d
Haenuki	0.082 ± 0.001 b
Tsugaruroman	0.068 ± 0.000 d
Aichinokaori	0.105 ± 0.008 b
Yuudai21	0.048 ± 0.001 e
Akitakomachi (Ibaraki)	0.121 ± 0.002 a
Akitakomachi (Chiba)	0.099 ± 0.003 b
Akitakomachi (Akita) A	0.117 ± 0.000 a
Akitakomachi (Akita) B	0.118 ± 0.007 a
Tsuyahime (Yamagata) A	0.057 ± 0.001 d
Tsuyahime (Yamagata) B	0.118 ± 0.001 a
Tsuyahime (Shimane)	0.086 ± 0.001 b
Tsuyahime (Miyagii)	0.098 ± 0.002 b
Koshihikari (Saga)	0.098 ± 0.003 b
Koshihikari (Ibaraki) A	0.104 ± 0.000 b
Koshihikari (Ibaraki) B	0.069 ± 0.001 c
Koshihikari (Shimane)	0.058 ± 0.002 d
Koshihikari (Niigata) A	0.060 ± 0.000 d
Koshihikari (Niigata) B	0.073 ± 0.001 c
Koshihikari (Yamagata) A	0.062 ± 0.001 d
Koshihikari (Yamagata) B	0.088 ± 0.000 b
Koshihikari (Ishikawa)	0.088 ± 0.000 b
Koshihikari (Yamanashi)	0.085 ± 0.001 b
Milkyqueen (Kyoto)	0.095 ± 0.001 b
Milkyqueen (Yamagata)	0.103 ± 0.003 b
Yumepirika (Hokkaidou) A	0.111 ± 0.000 a
Yumepirika (Hokkaidou) B	0.131 ± 0.004 a
